# Single-shot, depth-encoded multiplexed OCT for multi-spot tracking of induced transient corneal dynamics

**DOI:** 10.1364/BOE.596342

**Published:** 2026-04-29

**Authors:** Karol Karnowski, Jadwiga Milkiewicz, Onur Cetinkaya, Angela Pachacz, Ewa Mączyńska-Walkowiak, Patryk Młyniuk, Andrea Curatolo, Kamil Liżewski, Ahmed Abass, Dawid Borycki, Bartłomiej Kałużny, Susana Marcos, Ahmed Elsheikh, Ireneusz Grulkowski, Maciej Wojtkowski

**Affiliations:** 1 International Centre for Translational Eye Research, ul. Skierniewicka 10a, 01-230 Warsaw, Poland; 2 Institute of Physical Chemistry, Polish Academy of Sciences, ul. M. Kasprzaka 44/52, 01-224 Warsaw, Poland; 3 Institute of Physics, Faculty of Physics, Astronomy and Informatics, Nicolaus Copernicus University in Toruń, ul. Grudziadzka 5, 87-100 Toruń, Poland; 4 Department of Ophthalmology, Collegium Medicum, Nicolaus Copernicus University, ul. M. Curie Skłodowskiej 9, 85-094 Bydgoszcz, Poland; 5 Institute of Advanced Studies, Nicolaus Copernicus University, ul. Wileńska 4, 87-100 Toruń, Poland; 6 Department of Physics, Politecnico di Milano, Piazza Leonardo da Vinci 32, 20133 Milan, Italy; 7 Department of Materials, Design and Manufacturing Engineering, School of Engineering, University of Liverpool, Liverpool L69 3GH, UK; 8 Instituto de Óptica “Daza de Valdés”, Consejo Superior de Investigaciones Científicas, Madrid, Spain; 9 Center for Visual Science, The Institute of Optics, Flaum Eye Institute, University of Rochester, New York, USA

## Abstract

Fast, non-repeatable transient mechanical events in soft and scattering media are challenging to quantify because they demand high temporal bandwidth, high displacement sensitivity, and multi-point spatial coverage within a single realization. Current non-contact methods for assessing corneal biomechanics often rely on global metrics or single-meridian scanning, potentially missing the focal and asymmetric stiffness changes characteristic in corneal pathologies like keratoconus. In this work, we developed a simultaneous multi-spot air-puff optical coherence tomography (OCT) system as a generalizable parallel interferometric readout architecture to address the limitations of global metrics and single-meridian scanning in detecting focal corneal stiffness changes and to enable artifact-resistant measurement of rapid transients. By leveraging space-division multiplexing with depth encoding, our system tracks dynamic surface deformation at nine locations (one central, eight peripheral) simultaneously. This configuration eliminates sequential scanning artifacts and achieves an effective temporal resolution of 10 µs. We introduce the “Asymmetry Vector” to quantify the magnitude and direction of biomechanical imbalances. In experiments involving a keratoconus-mimicking phantom and human subjects (healthy and keratoconic), this vector correlated strongly with the specific location of corneal pathology. Furthermore, the system revealed a novel “dual-indentation” deformation profile resulting from a spatially widened air-puff stimulus. Beyond corneal elastography, the depth-encoded multiplexed OCT approach provides a scalable route to multi-point, high-speed characterization of transient dynamics where sequential scanning would otherwise induce spatiotemporal misregistration and waveform distortion. These findings establish the technical feasibility of simultaneous multi-spot OCT for biomechanical mapping without sequential-scanning-induced spatiotemporal misregistration and support its potential for automated clinical diagnosis.

## 1. Introduction

The precise characterization of transient, non-repeatable dynamic events in soft matter remains a central challenge for optical metrology. Many mechanically driven processes—ranging from high-frequency shear wave propagation to rapid viscoelastic response in biological tissue—unfold on sub-millisecond time scales and exhibit strong spatial heterogeneity [1–4]. Therefore, capturing such events requires modalities that provide (i) high displacement sensitivity, (ii) high temporal bandwidth, and (iii) multi-point spatial coverage within a single realization of the event.

Different strategies have been implemented to probe and visualize fast processes. Across imaging modalities, a common route to overcoming speed–resolution constraints is parallelization, in which multiple spatial channels are acquired simultaneously and separated either by hardware diversity (e.g., detector/receiver arrays) or by computational demixing (decoding) using known channel responses. Representative examples include Magnetic Resonance Imaging parallel imaging (e.g., SENSE and GRAPPA), Multi-Detector Computed Tomography, ultrafast (plane-wave) Ultrasound imaging, and Multi-beam scanning electron microscopy [5–9].

Conventional optical interrogation methods, however, face a fundamental architectural trade-off. Wide-field high-speed imaging provides highly parallel acquisition but often lacks the axial sensitivity and depth discrimination required to quantify micron-to-nanometer surface displacements in scattering media [10–12]. Conversely, interferometric ranging methods—such as swept-source optical coherence tomography (SS-OCT) and laser Doppler vibrometry—offer high axial displacement sensitivity, but spatial coverage is typically achieved by sequential (point) scanning [13–15]. However, this scanning paradigm becomes challenging for non-repeatable transients: the acquired dataset mixes the scanner trajectory with the object’s evolution, yielding spatiotemporal misregistration and potential aliasing of fast features. As a result, sequential acquisition can distort both the temporal waveform and its spatial distribution, which may affect attempts to infer local mechanical properties or to detect focal anomalies.

**Fig. 1.**
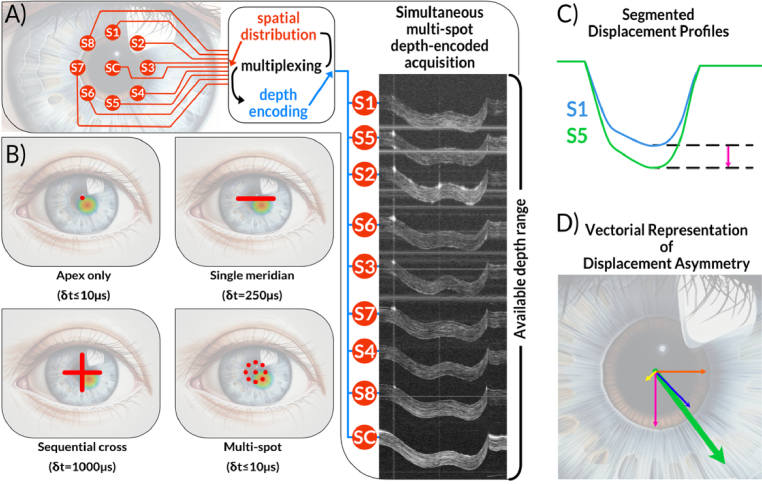
Current air-puff OCT scanning scenarios and concept of multi-spot air-puff OCT. A) graphical explanation of the spatial distribution of measurement points in the multi-spot air-puff OCT system with a central spot (SC) and 8 peripheral spots (S1-S8). This distribution (pace-division) combined with depth-encoding enables simultaneous measurements of corneal deformation dynamics at 9 distinct spots. Depending on the method used, the dynamic of air-induced corneal displacement is probed at limited cornea regions B) Representative air-puff OCT sampling scenarios. The apex-only, single-meridian, and sequential cross-meridian panels illustrate previously used or conventional acquisition strategies, whereas the bottom-right panel corresponds to the proposed multi-spot depth-encoded approach introduced in this work. C) Concept of differences in temporal corneal deformation profiles for two opposite peripheral plots (this is a concept, not profiles extracted from M-scan presented in A). D) Visualization of the asymmetry vector concept with the main asymmetry vector marked in green and directional asymmetry vectors marked in pink, yellow, orange, and blue for pairs S1 & S5, S2 & S6, S3 & S7, and S4 & S8, respectively. The circular-symmetric heatmap overlaid on images (B) represents the most frequent keratoconus cone occurrence [22].

Our strategy to remove these scanning-based artifacts is to parallelize interferometric readout through multiplexing (Fig. 1(A)). Space-division multiplexing (SDM), originally developed to increase channel capacity in optical communications, can be repurposed for optical metrology by interrogating multiple spatial locations simultaneously [16–18]. In SS-OCT, SDM can be realized efficiently using depth (path-length) encoding: distinct probe beams correspond to different optical delays so that their interference terms occupy non-overlapping depth windows within a single A-scan (Fig. 1(A) and (B) bottom-right). This approach exploits an extremely long instantaneous coherence length of modern swept sources, e.g. VCSEL-based lasers, as well as usable ranging depth that are large compared with the millimeter-scale depths of interest in many biomechanical experiments [19–21]. By allocating this available depth range across multiple spatial channels, depth-encoded SDM enables simultaneous multi-point tracking with a single detector channel, preserving point-detection sensitivity while providing spatial diversity in a single-shot acquisition (Fig. 1(A)).

Air-puff corneal elastography represents an example of this application. The cornea is a layered, viscoelastic shell whose mechanical symmetry can be compromised in ectatic diseases such as keratoconus (KC), where localized weakening often precedes clinical manifestation of morphological changes [23]. Air-puff excitation induces a rapid transient deformation (on the order of tens of milliseconds) and is a non-contact and clinically accepted procedure. However, established experimental implementations frequently rely on apex-only tracking or on a single meridians (Fig. 1(B)) [15,24–27]. Such sampling scenarios are inherently insufficient during probing focal, non-centrosymmetric biomechanics, and sequential scanning is additionally vulnerable to non-repeatability caused by fixation micro-motions and tear film dynamics [28–31]. Accordingly, those constraints motivate a measurement approach that can capture multi-point deformation dynamics simultaneously and with sufficiently high temporal resolution to preserve the transient waveform.

In this work, we present a simultaneous multi-spot air-puff OCT system that combines SDM with depth encoding to track dynamic corneal deformation at nine discrete locations (one central and eight peripheral – see Supplement 1. Spatial distribution of the sampling points) in a single acquisition. By mapping each spot to a dedicated depth band, the system eliminates sequential scanning artifacts while achieving an effective temporal resolution of 10 µs across all channels. Building on these measurements, we introduce an asymmetry vector metric that compresses inter-location differences in deformation (Fig. 1(C)) into vectorial representation (Fig. 1(D)) that quantifies the strength and orientation of biomechanical asymmetry. We demonstrate feasibility in a keratoconus-mimicking ex vivo model and in vivo measurements in healthy and keratoconic eyes, and we report a dual-indentation deformation profile observed with a spatially broadened air-puff stimulus. While the present study focuses on corneal biomechanics, the depth-encoded SDM-OCT architecture establishes a general route to artifact-resistant, high-speed characterization of non-repeatable transient mechanical events in scattering media.

## 2. Materials and methods

### 2.1. Multi-spot air puff OCT system

**Fig. 2.**
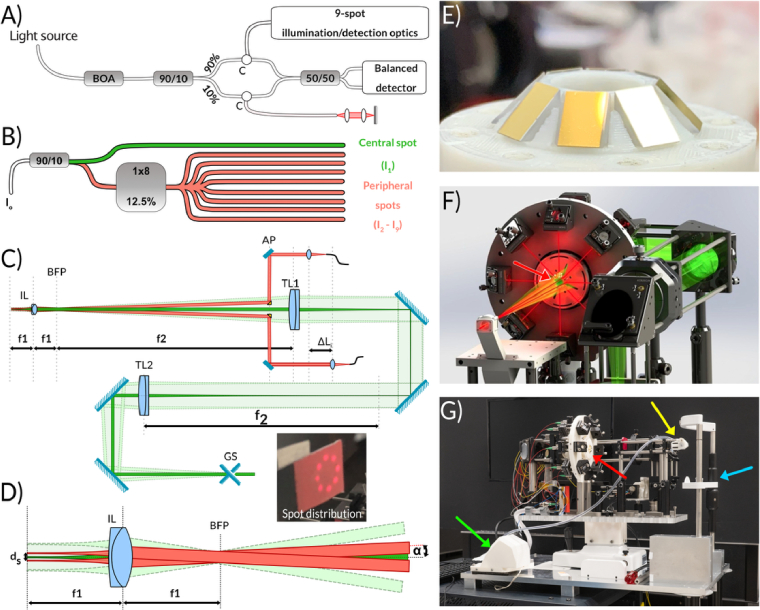
Optomechanical design of the system. A) Schematic of the fiber multi-spot swept-source OCT interferometer. C – circulator. B) Detailed scheme of the interferometer sample arm including 2 × 2 coupler (90/10 split ratio) and 1 × 8 coupler cascade. C-D) Optical configuration of the multi-spot air-puff OCT sample arm. The dark green path denotes the central beam during the final measurement. The light green path denotes the lateral scan range of the central beam during alignment (cross-hair OCT preview), and the green dotted-edge paths indicate the extreme scan positions. The red paths illustrate two opposite peripheral channels. The scan range is shown schematically and is not drawn strictly to scale. D) Zoom of the proximity of the imaging lens with the telecentric distribution of imaging beams. IL –imaging lens, BFP – the back focal plane of the imaging lens, TL1 and TL2 – telescope lenses, GS – galvo scanner system (Scanner Max, Saturn 5, Pangolin Laser Systems, Inc., Sanford, FL, U), AP – plane of the custom array, ΔLi – pathlength offset between channels, f_1_ and f_2_ – focal lengths of telescope lenses, d_s_ – separation between the opposite peripheral beams (d_s_ = 2*r), α – angle between peripheral beam and the optical axis of the system. Inset on C) – peripheral beams (collimated) distribution photographed on the IR viewing card. E) A photograph of a custom array of prism mirrors – a crucial part of peripheral beam alignment. F) 3D rendering of the optomechanical design with central and peripheral beams marked with colors corresponding to Fig. 1(A). G) A photograph of the clinical prototype with visible changes to the air-puff module compared to the initial design (rendered one). The green arrow on G) indicates the solenoid motor and piston module that was separated in the final design from the air-puff chamber head ( Supplement 1. Air-puff chamber) indicated with the yellow arrow. The blue arrow indicates the head/chinrest frame. The red arrows in F and G indicate the positions of the custom prism-mirror array.

The instrument is based on a fiber-optic swept-source OCT system. A frequency-tunable light source utilizing vertical-cavity surface-emitting laser technology (VCSEL, SL131090, Thorlabs Inc., Newton, NJ, USA) operates at the central wavelength of 1310 nm and at a sweep speed of 100 kHz. This light is amplified by a booster optical amplifier (BOA, Covega Corporation, Jessup, MD, USA) before entering an OCT interferometer (Fig. 2(A)). In OCT interferometer, the light is split between the sample and reference arms, with 90% of the optical power allocated to the sample arm and 10% to the reference arm (Fig. 2(A)). A configuration featuring a cascade of two fiber couplers is employed in the sample arm to generate all nine illumination spots (Fig. 2(B)). The first 2 × 2 coupler, with a 90/10 split ratio (TW1300R2A2, Thorlabs, Inc., Newton, NJ, USA), separates the central spot beam from the peripheral beams. Next, a 1 × 8 coupler (TDE1315HA, Thorlabs, Inc., Newton, NJ, USA), distributes the peripheral light to eight channels with even power distribution. The 90/10 split ratio of the first coupler provides the most uniform power distribution and collection efficiency.

In the clinical prototype, the power levels measured for the central spot and peripheral spots were 3.6 mW and 2.5 ± 0.2 mW, respectively, corresponding to a total power incident on the eye of approximately 24 mW. The beams can be treated as multiple independent sources for thermal-safety assessment, because the 1.1 mm center-to-center spacing of the peripheral spots exceeds the 1 mm thermal integration zone (limiting aperture), and therefore thermal summation does not apply [32]. During the patient-alignment phase, the ∼350 Hz cross-scan preview pattern (displayed at video rate in acquisition software) and manual positioning further distributed the thermal load over the corneal surface. For the 1310 nm operating wavelength, the limiting corneal maximum permissible exposure (MPE) during M-scan acquisition, when the beams are stationary, was calculated to be approximately 265 mJ/cm^2^. The radiant exposure of the central beam (3.6 mW), averaged over the 1 mm limiting aperture (0.00785 cm^2^), was 22.9 mJ/cm^2^, providing an approximately 11.5-fold safety margin relative to the MPE. Since the eight peripheral spots had lower power levels than the central channel, the overall multi-spot exposure remained below the applicable safety limit [33]. Retinal exposure was negligible because of the strong attenuation of 1310 nm radiation in the ocular media and substantial beam divergence at the posterior segment. [32]. Theoretically, a 90/10 split ratio should yield 12.5% more power in the peripheral channels; however, the measured values are attributed to power losses during beam propagation through the optical system, including the prism array assembly and losses at the edge of the exit aperture of the air-puff chamber. For the given power levels at the sample plane and with a neutral density filter (ND 3.0) placed in the sample arm, we measured a sensitivity of 109 dB for the central spot and an average of 100 dB for the peripheral spots.

To accommodate OCT signals from all nine channels in a single M-scan image, we leveraged the coherence properties of the vertical-cavity surface-emitting laser (VCSEL) technology (instantaneous coherence length of > 1 m) and a high sampling frequency digitizer. The laser is equipped with a k-clock signal generated with a 44-mm Mach-Zender interferometer delay, translating to an 11-mm OCT depth range. By utilizing the Dual-Edge Sampling feature of the digitizer (ATS9373, AlazarTech), we extended the effective depth range to 22 mm, with negligible sensitivity roll-off. The laser operates at a 100 kHz sweep rate, providing a temporal resolution of 10 μs for corneal displacement measurements.

### 2.2. 9-spot spatial distribution and temporal adjustment

The spatial light beam distribution illuminating the cornea was determined from the outcomes of biomechanical modeling of keratoconic and healthy corneas ( Supplement 1. Spatial distribution of sampling point). Finite element method modeling indicated that the maximum asymmetry in corneal displacement amplitudes for opposite spots for keratoconus cases could be observed at points 1.1 mm from the corneal apex. That conclusion was implemented in the design of light delivery set-up.

However, the derived distribution of sampling points on the cornea and proposed data acquisition strategy posed two main challenges: (i) delivering light to the sample plane, and (ii) enabling simultaneous measurement. Both challenges were addressed in the design of the system's imaging head. For practical reasons, the central beam was separated from the peripheral beams and was used both for pre-measurement eye alignment and for the final central-spot measurement. In Fig. 2(C)-(D), the dark green path represents the central beam position during the final measurement acquired after air-puff activation, while the light green path indicates the lateral scan range of the central beam used during the alignment procedure (see Supplement 1. Patient alignment). The green dotted-edge paths denote the extreme beam positions reached during this alignment scan. The red paths illustrate two opposite peripheral channels. The central scanning beam was delivered via a relay system (pair of lenses, TL1 and TL2; f_2_ = 300 mm, ACT508-300-C-ML, Thorlabs, Inc., Newton, NJ, USA) through an opening in the prism mirror array assembly. The peripheral beams were propagated via systems of mirrors (MRA05-M01, Thorlabs Inc.): one mirror per spot mounted on a kinematic mount for precise angle adjustment, and one fixed mirror from the custom prism mirror array (Fig. 2(E)). The peripheral beams were aligned to intersect at a given point in space along the optical axis of the system at an angle α (BFP in Fig. 2(C)-(D)).

In this configuration, with an imaging lens (IL in Fig. 2(C)-(D)) with a focal length of f_1_ placed at the back focal distance from this intersection point, a telecentric illumination with the peripheral spots on the corneal surface is expected, where the distance *d_s_* of the spot from the center is given by:

(1) $$d_{s}=f_{1}*\tan\alpha .$$

To maintain a small device footprint (the angle α is constrained by the aperture of the custom prism mirror array and its distance to the intersection point), we used an objective lens with focal distance f_1_ = 30 mm (AC127-030-C-ML, Thorlabs, Inc., Newton, NJ, USA), resulting in a 24 µm spot size in the focal plane. The beam distribution at the intermediate point (between the prism mirror assembly and the intersection point) is presented in Fig. 2(C) (inset). The alignment accuracy of the illumination spots in the sample plane was assessed from beam-profiler (WinCamD-UCD15, DataRay Inc., Monterey, CA, USA) measurements acquired at the focal plane. The central spot was taken as the reference point, and the intended peripheral-spot geometry was represented by a circle of 1.1 mm radius centered on this spot. Radial alignment error was calculated as the deviation of each peripheral spot from the nominal radius. Angular alignment error was determined by comparing the actual meridian defined by the line joining the central spot and a given peripheral spot with the corresponding ideal meridian of the intended 8-spot geometry. Using this procedure, the mean error of spot alignment in the sample plane was estimated to be 24 µm (maximum 60 µm) in terms of distance from the central spot, and 1.3° (maximum 2.4°) in terms of angular position along the selected meridians.

The central beam was scanned by a two-axis galvanometer system only during the alignment procedure, producing sequential horizontal and vertical preview B-scans, whereas the peripheral beams remained stationary. Consequently, in the preview images the central channel appears as a B-scan-like structure, while the peripheral channels appear as stationary point signals. During the final air-puff measurement, all nine beams were stationary; therefore, before the onset of corneal deformation, the corresponding OCT signals are speckle-free for all channels.

We also leveraged the depth range capabilities of modern SS-OCT systems to enable simultaneous measurement at all nine spots. With a 22-mm depth imaging range (in the air), we separated the OCT interferometric signal from each spot by introducing path-length offsets between illumination beams (ΔL_i_ in Fig. 2(C)). The minimum required offset is determined by the optical thickness of the cornea (corneal thickness multiplied by the refractive index of the cornea). Assuming the refractive index n = 1.337 and a maximum corneal thickness of 600 μm, an optical path length separation of ca. 2 mm would be sufficient.

The short focal length of the objective lens (IL in Fig. 2(C)-(D)) has several practical consequences. First, it limits the lateral scan range available for cross-sectional OCT preview; in the current implementation, the usable preview scan width was approximately 6 mm. Second, it reduces the depth of focus. For the 30 mm focal-length imaging lens used here, the focused spot diameter in the sample plane was approximately 25 µm, corresponding to a depth of focus of about 0.72 mm.

In the context of the present 9-spot geometry, this depth of focus is sufficient to ensure comparable imaging conditions for the central and peripheral corneal locations. Assuming a corneal radius of curvature of 7.8 mm and peripheral sampling points located 1.1 mm from the apex, the axial height difference between the apex and a peripheral point is approximately 79 µm, which is nearly one order of magnitude smaller than the depth of focus. Therefore, the corneal curvature over the sampled region does not lead to substantial differences in focusing conditions between channels.

Because the present analysis was restricted to temporal tracking of the anterior corneal surface at fixed sampling locations, explicit correction of OCT images for corneal curvature and refraction was not required for the extracted displacement parameters. In this context, the most critical factor was stable and repeatable beam placement. The central beam was aligned close to normal incidence at the corneal apex, while the peripheral beams were arranged in a telecentric geometry and individually adjusted using kinematic mirrors. Residual deviations were quantified through the spot-placement analysis described above.

At the same time, the relatively shallow depth of focus is advantageous in this application, because it helps restrict signal collection primarily to the cornea and reduces contributions from deeper ocular structures, such as the iris or crystalline lens, which could otherwise complicate the interpretation of the depth-encoded multi-channel OCT signal. Finally, the small footprint of the 30 mm lens facilitated miniaturization of the air-puff chamber and its integration with the optical setup.

Finally, the small footprint of the 30 mm lens enabled smooth miniaturization of the air-puff chamber and its integration with the optical setup. The optomechanical components of the imaging head (depicted in Fig. 2(F)) were mounted on a small plastic breadboard (20 × 48 cm), making it compact enough for use with the clinical joystick-chinrest platform (Fig. 2(G)).

### 2.3. In vivo measurements

In vivo measurements using the clinical prototype (Fig. 2(G) and Fig. S3A) were conducted at the Ophthalmology Clinic (OFTALMIKA, Bydgoszcz, Poland) in adherence to the Declaration of Helsinki and with the approval from the Ethics Committee at the Collegium Medicum, Nicolaus Copernicus University (KB 689/2021). Following informed consent, the eyes of thirteen healthy subjects volunteers and three KC patients—were first examined using Placido disc topography and high-resolution anterior segment OCT (MS-39, CSO, Italy). Subsequently, the air-puff-induced corneal response was assessed with the multi-spot air-puff OCT prototype following a careful patient alignment procedure ( Supplement 1. Patient alignment). Alignment quality was verified from the last OCT preview acquired immediately before air-puff activation; measurements showing evidence of misalignment were excluded from further analysis and reacquired.

### 2.4. Data visualization strategies

**Fig. 3.**
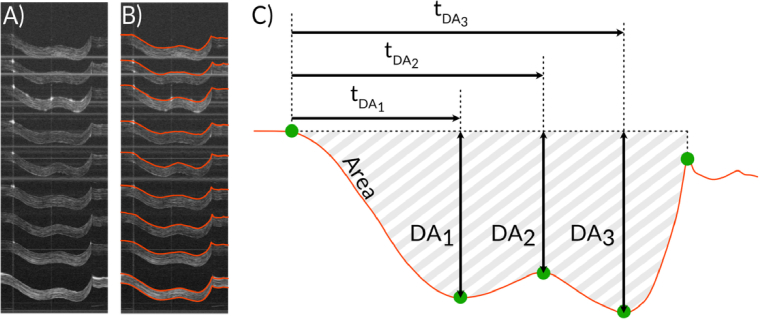
Extracting corneal displacement profiles. A) Representative 9-spot corneal displacement M-scan. B) Displacement M-scan with segmented temporal displacement profiles for each of the imaging spots overlayed in red. C) Parameters extracted from displacement profiles and used for further analysis: displacement amplitudes DA_1_-DA_3_, time between displacement start and given amplitude t_DA1_-t_DA3_, and the area enclosed by the displacement curve (grey pattern region).

Manual segmentation of the anterior corneal surface at each of the nine imaging spots was used to convert the tracked boundary into displacement-versus-time profiles, from which nine deformation parameters were extracted. Manual segmentation (delineation of the anterior cornea displacement) was performed on all images using open-source software (GIMP). The process involved adding a mask layer on top of the processed OCT M-scan (Fig. 3(A)). Then a curve (red overlay in Fig. 3(B)) was drawn on the mask layer following the anterior-corneal-surface temporal displacement for visual inspection of the delineation accuracy. The corresponding mask was saved as a png-file for each measurement. Mask files were loaded into a MATLAB script and converted to displacement data, from which the displacement parameters were extracted (Fig. 3(C)).

We focused on 9 parameters extracted from displacement profiles: displacement amplitudes DA_1_, DA_2_, and DA_3_, the mean values (DA_1_ + DA_3/2_) of displacement amplitude, DA1/DA_3_ ratio, time between displacement start and given amplitude t_DA1_, t_DA2_ and t_DA3_, and finally the area enclosed by the displacement curve (Fig. 3(C)).

**Fig. 4.**
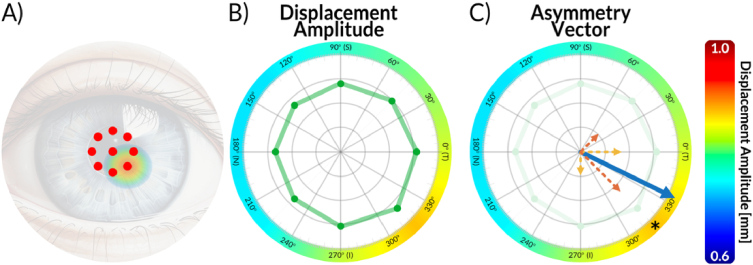
Data processing and results visualization. A) Photo of an eye with a 9-spot distribution overlay, along with the most frequent occurrence of keratoconus cones (the circular heat map region). B) Polar plot visualization of the dynamic biomechanical asymmetry in the air-induced corneal displacements extracted from randomly selected eyes within the keratoconus group. The green points represent the displacement amplitude (extracted from M-scans) connected with a solid green line. C) Visualization of the asymmetry vector for the same keratoconus patient. Dotted arrows indicate the asymmetry vectors for each opposite peripheral spots pair. The solid-line arrow represents all vectors combined (blue). The color rings overlay on B)-C) provide an additional visualization method, generated from asymmetric shapes (green shapes in B) and E)) followed by spline interpolation for smoother color transition. For comparison, an asterisk indicating the maximum displacement values for peripheral points has been added in C).

Displacement parameters are plotted in polar coordinates (here using Matlab’s polarplot) at angular positions matching each spot on the corneal surface (Fig. 4(A)-(B)). An asymmetric distribution may indicate corneal biomechanical asymmetry (Fig. 4(B)). The standard 8-point polar plot is enhanced with a peripheral heat-map ring: the 8 discrete points show raw values at peripheral spots, while the surrounding annulus uses angular interpolation to generate a smooth color gradient. Color temperature maps directly to the polar values, emphasizing maxima and minima; in Fig. 4(B)–(C), the color-map maximum occurs at an azimuth of 315°.

To move beyond qualitative visualization of the peripheral deformation pattern, we defined an asymmetry vector as a compact descriptor of the directional imbalance in the amplitudes of opposite-pair displacements. In this representation, the vector magnitude quantifies the strength of the asymmetry, while the vector angle indicates its dominant orientation. Displacement amplitudes from opposing corneal locations were paired to form meridional asymmetry vectors: vector length represents the absolute inter-pair difference, and direction points toward the higher-displacement location. Those vectors are shown as dotted arrows in Fig. 4(C): orange denotes cardinal (horizontal/vertical) meridians, and red denotes intercardinal (45°/135°) meridians. Vector summation yields an overall asymmetry vector (solid blue arrow, Fig. 4(C)). Unlike the polar plot, which preserves the full spatial distribution but remains descriptive, the asymmetry vector compresses the multi-spot response into a single quantity that can be readily compared across eyes and repeated measurements.

Here, the asymmetry vector points downward, indicating the region with the largest displacement. This directionality, consistent with prior reports [15,24,25,34], likely reflects a biomechanically weaker or thinner area, as typically observed in KC. Its orientation differed slightly from the heat-map maximum (asterisk, Fig. 4(C)), since the color map is based on spline-interpolated peripheral values for smoother display. The asymmetry vector magnitude can serve as a quantitative metric for statistical analysis and biomarker evaluation.

Although several deformation-derived parameters were evaluated, DA_1_ was chosen as the principal parameter for the main analysis because it showed the most robust combination of vector-magnitude discrimination and directional consistency in the present dataset; the behavior of the additional parameters is summarized in Supplement 1. Preselection of optimal parameter of corneal displacement.

## 3. Results

We present the evaluation of the proposed method in three stages. First, we characterize the conditions under which our system reveals the air-puff–driven dual-indentation response and summarize its key spatiotemporal features (Section 3.1). Next, we assess sensitivity to focal biomechanical changes by detecting localized softening in an ex vivo model (Section 3.2), and we then perform a comparative analysis of biomechanical asymmetry in human subjects (Section 3.3). Finally, we quantify measurement repeatability (Section 3.4) and analyze how temporal sampling rate affects the estimated asymmetry (Section 3.5), confirming that sufficiently high temporal resolution is required for reliable characterization of these transient deformation dynamics. Results can be presented either in polar coordinates or in the form of an asymmetry vector for a range of parameters derived from the corneal displacement profiles. Among these, we identified the primary displacement amplitude (DA_1_) as the most informative metric, as its asymmetry vector most sensitively reflects focal corneal changes. Accordingly, all results presented here are restricted to DA_1_.

### 3.1. Complex air-puff induced corneal dynamics

To preserve OCT image quality for the peripheral probe beams, we customized the air-puff chamber geometry. Specifically, we enlarged the chamber exit aperture to mitigate vignetting and the associated signal loss at off-center imaging locations. In this study, we selected a 3.7-mm exit aperture (vs. 1.5 mm in the original design), which was the smallest diameter that maintained stable imaging performance for the peripheral beams. This modification produced a spatially broader air stimulus (see Supplement 1. Air-puff chamber) and reduced its peak strength by approximately twofold relative to the commercial device.

**Fig. 5.**
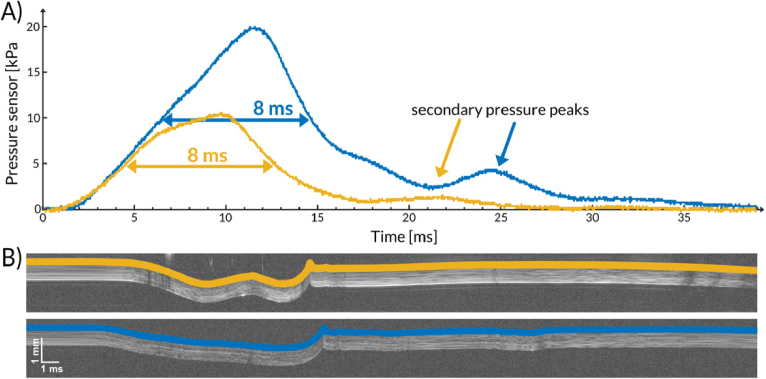
Impact of air-pulse stimulus customization. (A) Temporal profiles of the air-puff stimulus measured for the original and customized exit apertures (blue: 1.5-mm bore; orange: 3.7-mm bore). (B) Air-puff-induced corneal displacement at the apex measured by OCT in the same human subject for the two exit-aperture configurations shown in (A). Panels A and B use the same temporal scale and observation window, but the pressure waveform and corneal deformation data were acquired separately; therefore, the relative time-zero alignment is approximate and intended for qualitative comparison only.

Despite the pressure reduction (Fig. 5(A); blue: original aperture, orange: customized aperture), the temporal width of the pulse remained unchanged (8 ms FWHM). Notably, the difference in corneal displacement amplitudes was even more pronounced for the customized aperture (Fig. 5(B); orange vs. blue). Importantly, the customized stimulus elicited a novel dual-indentation response—two temporally distinct displacement events within a single pulse—that, to our knowledge, has not been reported previously (Fig. 5(B), orange trace).

Secondary pressure peaks are occasionally observed (Fig. 5(A)). However, these features are substantially weaker (typically >4× lower in amplitude than the primary peak) and occur later in time. By the time the secondary peaks arrive, the cornea has largely recovered to its baseline position, and the measured displacement is increasingly dominated by slower, non-corneal motion components—most notably globe retraction associated with the onset of eyelid closure. Consequently, these secondary peaks are unlikely to contribute meaningfully to the primary corneal deformation response and are treated as negligible for the analysis presented here.

Figure 5(A) and 5(B) are shown on the same temporal scale and over the same observation window, which enables qualitative comparison of the air-puff waveform and the corneal deformation response. However, because the pressure trace and OCT data were not acquired simultaneously in the present implementation, the panels should not be interpreted as an exactly synchronized stimulus-response measurement. Within this qualitative comparison, the secondary pressure feature visible in Fig. 5(A) does not appear to produce a clearly distinguishable or comparably strong secondary corneal deformation event in Fig. 5(B).

### 3.2. Control tests in ex vivo porcine eyes

**Fig. 6.**
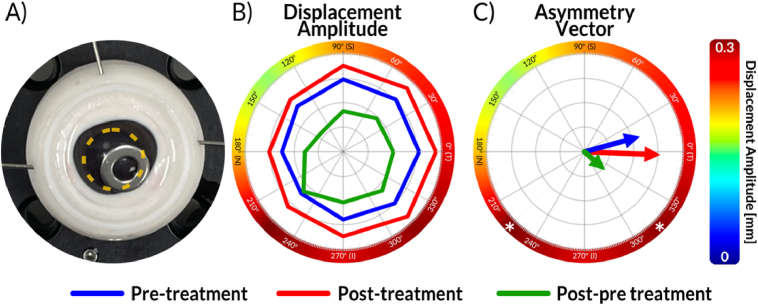
Evaluation on keratoconus phantom. A) Photograph of an ex vivo porcine eye with a visible metal washer (removed prior to air-puff OCT measurements). The yellow-dashed circle represents the eye’s pupil. B) Polar plot shapes derived from measurements taken before (blue) and after (red) treatment with liberase. The green shape represents the difference obtained by subtracting the pre-treatment shape. C) Asymmetry vectors extracted from measurements before (blue) and after (red) treatment, with a green vector representing the difference. The outer color ring overlayed on B)-C) provides an additional visualization method, generated from asymmetric shapes (green shapes in B) followed by *spline* interpolation for smoother color transition. For comparison, an asterisk indicating the maximum displacement values for peripheral points has been added in C). Please note that two such maxima are present in C), which would make assessment challenging; however, the asymmetry vector provides a clear indication of corneal asymmetry.

We developed a keratoconus-mimicking phantom by chemically softening the lower-right section of the cornea of porcine to demonstrate the system's capability to detect localized abnormalities in corneal structure (Fig. 6(A) and Supplement 1. Keratoconus phantom).

For the phantom experiments, whole porcine eyes obtained post mortem from a certified local abattoir were used. Biological-material handling and disposal were performed in accordance with the established procedures of the collaborating laboratory. The globes were mounted in a dedicated 3D-printed ocular holder, and intraocular pressure was maintained at 15 mmHg using a water-column system connected via a needle inserted into the posterior chamber. Localized biomechanical weakening was induced by topical application of Liberase HI (20 mg/ml) mixed 1:1 with 30% dextran, with the treatment area confined by a metal washer, as described in the Supplement 1.

To isolate the effect of localized corneal tissue weakening, we subtracted the pre-procedure asymmetry profile from the post-procedure measurement. The resulting polar plot (Fig. 6(B), green) shows two distinct maxima at 315° and 225° (white asterisks in Fig. 6(C)), making it difficult to precisely assess the direction of the treatment-induced changes. However, using the asymmetry vector approach, the resulting vector (green arrow in Fig. 6(C)) points in the direction where the enzyme was applied (the area enclosed by the washer in Fig. 6(A)). The angle of the green asymmetry vector was measured to be 318°, closely matching the azimuth angle (321°) which corresponds to the position of the center of the washer with respect to the center of the pupil of the porcine eye (as seen in Fig. 6(A)).

The asymmetry vector representation proved useful because it reduced the multi-point displacement pattern to a single directional descriptor, facilitating comparison between measurements and enabling direct assessment of whether the dominant asymmetry pointed toward the softened or clinically abnormal corneal region.

### 3.3. In vivo measurements of healthy subjects and keratoconus patients

**Fig. 7.**
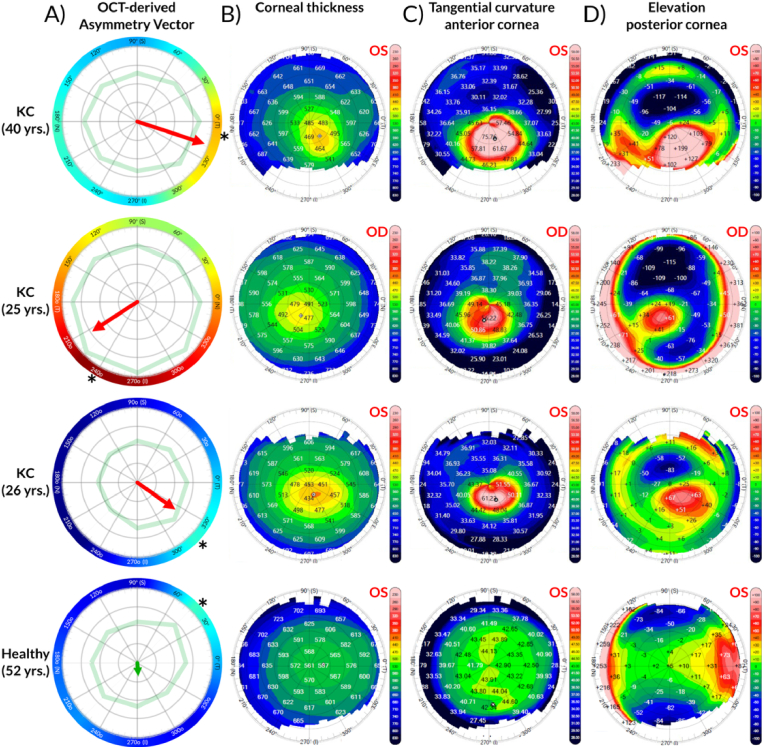
Comparison of proposed qualitative visualization with corneal maps from the commercial device. A) Asymmetry vectors plotted for 3 examples of keratoconus patients (for 3 rows) and one example of a healthy person for comparison. B-D) maps provided by the commercial device (MS-39 system form CSO) covering corneal thickness, tangential curvature of anterior corneal surface, and elevation of the posterior corneal surface respectively. The asymmetry vector in A) is colored red for KC patients, or green for healthy subjects. Grey vectors are added for reference in column A) and represent asymmetry vectors for 4 additional healthy patients. Additionally, a light green shape reflects the values of the displacement amplitude at each peripheral spot.

The diagnostic capability of the multi-spot air-puff OCT system is demonstrated in Fig. 7, which compares the proposed asymmetry-vector-based outcome (Column A) with standard corneal tomographic maps (Columns B–D) across three KC cases and one healthy control. A clear distinction in vector magnitude is observed: KC eyes (top three rows) exhibit significantly longer asymmetry vectors (red arrows) compared to the minimal vector length seen in the healthy eye (green arrow, bottom row). This observation reflects the pronounced biomechanical and geometrical imbalances inherent to ectatic corneas.

Furthermore, the orientation of the asymmetry vector shows a strong spatial correspondence with pathological changes identified by the reference corneal topography maps (corneal thickness, corneal curvature, and elevation). In all KC subjects, the vector points consistently toward the “cone” region—characterized by localized thinning (Corneal Thickness, Column B), steepening (Tangential Curvature, Column C), and abnormal elevation (Elevation Posterior Cornea, Column D). While the spatial overlap is not absolute—likely because the air-puff response integrates both geometrical factors (thickness) and local biomechanical properties (stiffness)—the directional agreement validates the system's ability to localize abnormalities. This suggests that the multi-spot approach successfully captures the complex interplay of morphological and mechanical asymmetry distinctive to KC.

To provide a quantitative comparison with the clinical reference, we measured the angular position of the asymmetry vector and compared it with the angular location of the abnormal sector derived from the MS-39 corneal maps. For the three keratoconus eyes shown in Fig. 7, the asymmetry-vector angles were −18.3°, −146.7°, and −35.6°, respectively. The corresponding angular positions obtained from the commercial device were: for the first KC patient (40 yrs.), −51.8° from the pachymetry map, −70.8° from the anterior tangential curvature map, and −58.3° from the posterior elevation map; for the second KC patient (25 yrs.), −123.4°, −105.9°, and −120.7°; and for the third KC patient (26 yrs.), −65.7°, −66.3°, and −47.5°, respectively. These values indicate directional agreement, but not exact overlap, between the proposed asymmetry vector and the clinical geometric reference.

### 3.4. Repeatability of the asymmetry vector

**Fig. 8.**
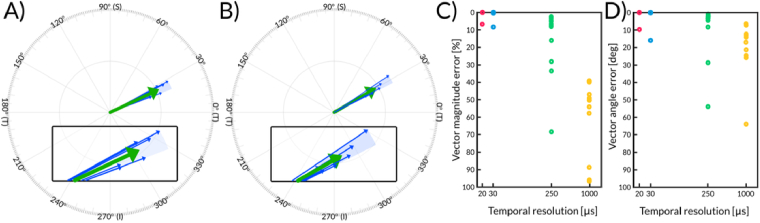
A-B) Repeatability of the asymmetry vector for the displacement amplitude. (A, B) Polar plots showing repeated acquisitions (thin blue arrows) relative to the mean asymmetry vector (thick green arrow) for two different healthy eyes. Insets in (A, B) represent magnified views of the vector tips from panels A and B, respectively. The light blue shaded regions represent the standard deviation of the vector alignment. The close proximity of the individual blue vectors to the mean green vector illustrates the low variability in both angle and magnitude across repeated measurements. C-D) Impact of temporal resolution on measurement results. The analysis was performed using effective temporal resolutions of 20 μs, 33.3 μs, 250 μs, and 1 ms, derived from thirteen eyes (ten healthy and three keratoconus (KC)) measured with our system. Results from the native 10 μs resolution serve as the reference. C) The maximum error in calculated asymmetry vector amplitude, expressed as a percentage of the full resolution (10 μs) data. D) Angular errors for asymmetry vectors. Marker colors in C–D correspond to the simulated temporal resolutions (20 μs: red, 33.3 μs: blue, 250 μs: green, 1 ms: yellow).

The repeatability of the system is visualized in Fig. 8(A)-(B), which displays the asymmetry vectors for displacement amplitude values obtained from two healthy volunteers. For each eye, a minimum of five properly aligned repeated measurements were acquired within a single session, with repositioning and re-alignment performed between acquisitions. Measurements that did not satisfy the alignment criteria, described in Supplement 1. Patient alignment, were excluded and repeated. In the asymmetry-vector polar plots (Fig. 8(A)-(B)), the individual measurements (represented by thin blue arrows) cluster tightly around the calculated mean vector (thick green arrow), indicating high repeatability.

The magnified insets (Fig. 8(C)-(D)) provide a detailed view of the asymmetry vectors, highlighting the spatial distribution of the acquisition errors. The light-blue-shaded area corresponds to the spread of asymmetry vectors across individual measurements. Quantitatively, this analysis revealed error ranges of 2.7–3.7 degrees in angle and 5–8% in normalized vector magnitude. Specifically, for the first eye (Fig. 8(A)), the standard deviation was 3.7 degrees for the angle and 4.3 pixels for the vector magnitude (corresponding to 37 µm, or a 5% normalized error). For the second eye (Fig. 8(B)), the standard deviations were 2.7 degrees, 7.3 pixels (equivalent to 63 µm), and 0.08 (8%) for the normalized vector magnitude. The visual overlap of the blue vectors and the narrow dispersion ranges shown in the zoomed views underscores the system’s robustness in generating consistent measurements for studying subtle corneal biomechanical asymmetries.

### 3.5. Impact of the temporal resolution on asymmetry vector quantification

We also evaluated the impact of temporal resolution—defined as the time interval between consecutive A-scans—on the accuracy of corneal deformation measurements. Our system operates at a temporal resolution of 10 µs, determined by the 100 kHz sweep rate of the swept laser. To benchmark this against lower-speed configurations, we numerically down-sampled the measured data (see Supplement 1, Temporal down-sampling) to simulate temporal resolutions of 20 µs, 33.3 µs, 250 µs, and 1 ms. [15,28,35,36].

We calculated relative errors for both vector magnitude and vector absolute angle for all possible down-sampling cases and for given eye, and temporal resolution plotted the biggest error (Fig. 8(C)-(D)). The results revealed a strong dependence of asymmetry-vector accuracy on temporal sampling. Relative to the full-resolution 10 µs dataset used as the reference, simulated down-sampling to 20 µs and 33.3 µs produced negligible vector-magnitude errors, whereas 250 µs sampling produced errors up to 20.0%, and 1 ms sampling produced errors approaching 70%. Similarly, the vector-angle error increased with coarser temporal sampling, reaching values up to 17.5° for 250 µs and 47.5° for 1 ms.

For each simulated temporal resolution, the reported error corresponds to the largest deviation observed across the possible temporal offsets of the down-sampled grid relative to the original 10 µs measurement. Therefore, these values should be interpreted as worst-case errors caused by temporal phase mismatch between the sampling grid and the transient corneal deformation, rather than as repeatability statistics.

## 4. Discussion and conclusions

In this work, we presented a multi-spot air-puff OCT platform that combines space-division multiplexing, depth encoding, and a custom designed air-pulse module to enable simultaneous monitoring of dynamic processes such as corneal deformation at nine locations with microsecond temporal resolution. On top of this acquisition scheme, we introduced an asymmetry-vector representation that condenses differences in displacement amplitudes between peripheral locations into a single magnitude-and-direction metric. In contrast to conventional air-puff techniques that provide apex-only or single-meridian measurements, the proposed approach directly targets the non-centrosymmetric biomechanics that are characteristic of KC and other ectatic disorders. From an optical metrology standpoint, the key advance is the use of the OCT ranging depth as a multiplexing resource: controlled path-length offsets assign each spatial channel to a distinct depth window, enabling simultaneous readout without multiplying detector hardware. This architecture specifically addresses the spatiotemporal misregistration inherent to sequential scanning of non-repeatable transients.

The emphasis of this manuscript is on method development and feasibility rather than on exhaustive clinical validation. To that end, we combined engineering characterization of the air-pulse and imaging head with proof-of-concept demonstrations in a keratoconus-mimicking phantom and a small cohort of KC patients and healthy subjects. Across these experiments, the asymmetry vector consistently highlighted regions of increased deformation, with orientations that coincided with areas of localized thinning and steepening in topographic maps and with the chemically weakened region in the phantom. These results support the idea that combining spatially multiplexed measurements with a compact vector representation can provide an intuitive map of biomechanical imbalance using a single, short acquisition.

The phantom experiment clearly illustrates this advantage. When the lower-right quadrant of a porcine cornea was softened enzymatically, the difference polar plot obtained by subtracting pre- from post-treatment displacement amplitudes exhibited multiple maxima, making it difficult to visually identify the treated sector. In contrast, the asymmetry vector derived from the same data pointed directly toward the softened region, with only a small angular difference compared to the known position of the washer. This demonstrates how the vector operation integrates information from all peripheral locations and suppresses local fluctuations that might otherwise obscure the primary axis of biomechanical change.

We also quantified the repeatability of the asymmetry vector in healthy volunteers. Multiple measurements per eye showed small dispersion in both angle and normalized magnitude, corresponding to angular standard deviations on the order of a few degrees and magnitude variability of 5–8%. These results indicate that, under appropriate alignment ( Supplement 1. Patient alignment), the multi-spot system provides stable measurements of corneal biomechanical asymmetry. Such repeatability is essential if the method is to be used for longitudinal monitoring or for detecting subtle changes, for example after corneal cross-linking or refractive surgery. The present repeatability assessment was designed to evaluate measurement consistency under controlled single-session conditions. It does not capture longer-term physiological sources of variability, such as inter-session differences or diurnal fluctuations in intraocular pressure, which may also affect the corneal air-puff response and should be addressed in future studies.

The asymmetry vector should be interpreted as a derived summary metric rather than merely a graphical convenience. Its magnitude reflects the net strength of directional asymmetry in the peripheral deformation response, and its angle reflects the dominant axis of imbalance. While polar plots and individual opposite-pair differences remain informative and preserve more local detail, they are less convenient for direct comparison across subjects, repeated acquisitions, or experimental conditions. The asymmetry vector therefore provides a compact and reproducible way to summarize the spatially distributed response in a form suitable for repeatability analysis and future classification-oriented studies.

Exact angular agreement between the asymmetry vector and the clinical topographic maps should not be expected. The MS-39 maps primarily characterize corneal geometry, whereas the proposed air-puff OCT metric reflects the corneal dynamic response, which depends on both geometry and biomechanical properties. For example, previous studies have shown that increased corneal thickness and increased stiffness both reduce displacement amplitude [15,24,25], whereas in keratoconus, the co-localization of thinning and weakening tends to increase local displacement. Therefore, the asymmetry vector is expected to identify the general direction of the abnormal corneal sector, but not necessarily to coincide exactly with any single geometric map. Because the present keratoconus cohort was limited to three eyes, the study was not designed to support formal statistical analysis of angular agreement or diagnostic performance. Accordingly, the present results should be interpreted as methodological proof-of-concept rather than clinical validation.

It must be emphasized that the air-puff-induced corneal displacement measured here does not provide a direct estimate of intrinsic biomechanical parameters such as stiffness or viscoelasticity. The corneal response to an air-puff stimulus is a complex physical process influenced by multiple factors, including corneal geometry, intraocular pressure, boundary conditions, and tissue mechanical properties. Therefore, no direct analytical relationship between the measured displacement amplitudes and specific biomechanical parameters is claimed in the present work. At the same time, spatial asymmetry in the displacement response is expected to reflect underlying mechanical heterogeneity at least qualitatively. In particular, regions that are thinner and/or mechanically weaker are expected to deform more under the same loading conditions, which provides a plausible basis for relating the asymmetry vector to focal biomechanical abnormality. A rigorous recovery of intrinsic biomechanical markers, however, would require dedicated inverse modeling, for example using finite-element methods, and is beyond the scope of the present methodological study.

The system’s temporal tissue deformation sampling capability (10 µs) provided a unique opportunity to quantify the dependence of the asymmetry vector on acquisition speed. By numerically down-sampling the experimental datasets to emulate slower devices, we demonstrated that resolutions of 250 µs and 1 ms can yield large deviations in both vector magnitude and direction. In several instances, these errors were sufficient to cross the decision thresholds established by receiver operating characteristic analysis. Consequently, reduced temporal sampling appears to not only underestimate total deformation but also distort the observed spatial distribution, with clear implications for diagnostic classification. This observation illustrates a fundamental limitation for any method attempting to characterize spatially resolved biomechanics from fast transient events. These results are consistent with a spatiotemporal under-sampling effect: when the deformation contains fast acceleration phases, coarse temporal sampling biases both peak timing and amplitude, which then propagates into spatial asymmetry metrics.

The custom designed air-puff module was central to enabling multi-spot measurements. Separating the chamber head from the piston and integrating an anti-reflection-coated objective lens improved optical throughput, especially for peripheral beams, and reduced vibrational coupling into the imaging head. At the same time, widening the exit nozzle, which was necessary to avoid clipping the peripheral OCT beams, substantially altered the spatial distribution of the air pulse ( Supplement 1. Air-puff chamber). Direct measurements of the air-pulse using a high-speed pressure sensor showed that, as the nozzle diameter increased, the peak pressure decreased and the temporal full-width at half-maximum remained approximately constant, while the spatial full-width at half-maximum broadened from a few millimetres to several millimetres.

Several limitations of the current study should be acknowledged. First, regarding the observed dual-indentation phenomenon, a comprehensive biomechanical analysis of this phenomenon is beyond the scope of this technical report and will be the subject of future dedicated work. At present, we interpret the dual-indentation profile as a corneal response elicited under the modified air-puff conditions used in this study, particularly the broadened spatial loading profile. Direct pressure measurements showed that increasing the nozzle diameter substantially broadened the spatial full-width at half-maximum (FWHM) of the stimulus, while the temporal FWHM remained approximately constant at ∼8 ms. Under these conditions, the corneal response changed from a single-indentation profile to a two-peak deformation pattern. This suggests that the phenomenon may reflect an interaction between the broadened loading geometry and the intrinsic biomechanical response of the cornea, potentially including viscoelastic effects. However, the present data do not allow us to quantitatively relate the peak separation to a specific relaxation time, and a rigorous mechanistic interpretation will require dedicated future study, likely supported by finite-element modeling.

Second, we emphasize that the current manuscript provides a methodological foundation and feasibility demonstrations rather than a definitive clinical validation study. Accordingly, the focus is on the depth-encoded multiplexed OCT approach and the associated multi-spot deformation metrics. A dedicated clinical validation dataset has been collected under the same protocol, including control, keratoconus, and forme fruste keratoconus groups, and will be reported in a separate manuscript addressing diagnostic performance and clinical covariates.

Third, in this technical work we opted for a manual segmentation workflow to extract displacement profiles from M-scans. This approach is transparent, allows careful visual inspection of the delineated corneal boundary, and was sufficient for the relatively modest dataset analysed here. However, manual processing is time-consuming and potentially observer-dependent. Automated segmentation methods, including machine-learning-based algorithms developed on the same datasets, are currently under our active investigation and will be presented in a separate report. These approaches are expected to reduce processing time and improve reproducibility, thereby better matching the requirements of large-scale clinical deployment.

Finally, the current optical design—with a short focal length objective lens—prioritizes isolation of corneal signals and compactness of the imaging head at the expense of depth of focus. As a result, precise patient alignment is critical: small changes in eye position can markedly affect both signal quality and the effective sampling locations. We mitigated this through a dedicated alignment procedure based on central-spot preview scans and by rejecting misaligned acquisitions, but further improvements, such as integration of real-time alignment feedback or eye tracking, might be important to increase robustness in routine clinical environments.

While the current implementation relies on intensity-based segmentation of the anterior corneal surface, phase-resolved OCT approaches could provide enhanced displacement sensitivity and enable direct estimation of strain via differential phase measurements between corneal interfaces. However, implementing such methods in a multiplexed, depth-encoded, single-shot acquisition framework presents additional challenges related to phase stability, inter-channel phase consistency, motion-induced phase decorrelation, and reliable phase unwrapping. Exploration of such phase-sensitive extensions is therefore an important direction for future work.

The performance of the system is sensitive to alignment and subject stability. The main failure modes include incomplete centering of the multi-spot pattern relative to the corneal apex, involuntary eye movements, and blinking. These factors can reduce signal quality, alter the effective spatial sampling positions on the cornea, and consequently bias both the magnitude and direction of the asymmetry vector. In the present study, such effects were mitigated through the preview-based alignment procedure described in Supplement 1. Patient alignment and acquisitions showing evidence of misalignment were excluded and repeated.

Future versions of the system could benefit from integration of an iris (pupil) monitoring camera placed at a pupil-conjugate plane and combined with the existing OCT preview. Such a module, for example, implemented between TL2 and the galvo scanners using a dichroic beam splitter, could improve alignment robustness, reduce operator dependency, and facilitate more automated clinical operation.

While the present study focuses on asymmetry in corneal biomechanics, the depth-encoded SDM-OCT architecture provides a scalable and artifact-resistant framework for high-speed characterization of non-repeatable transient mechanical events in scattering media, extending beyond the specific ophthalmic application demonstrated here.

In conclusion, this work demonstrates that simultaneous multi-spot air-puff OCT, combined with an asymmetry-vector representation, can provide repeatable, spatially resolved measurements of corneal biomechanical asymmetry and can qualitatively localize keratoconic cones in vivo. More broadly, depth-encoded SDM provides a general interferometric strategy for single-shot measurement of transient, non-repeatable dynamics when sequential scanning would otherwise impose aliasing or misregistration. The system’s high temporal resolution and custom designed air-puff module enable new insights into the dynamics of corneal deformation, including the discovery of a dual-indentation response associated with spatially broadened pressure stimuli. Overall, the proposed approach provides a technically feasible route toward spatially resolved, non-contact assessment of corneal biomechanics. Future work will integrate automated segmentation and analysis pipelines and will leverage larger clinical cohorts to rigorously evaluate the diagnostic performance of asymmetry-based metrics for keratoconus detection, risk stratification, and treatment monitoring.

## Supplemental information

Supplement 1 Supplement 1 https://doi.org/10.6084/m9.figshare.32038821

## Data Availability

Data underlying the results presented in this paper are not publicly available at this time but may be obtained from the authors upon reasonable request.

## Supplemental document

See Supplement 1 for supporting content.
