# Supplementary material for: Single-shot, depth-encoded multiplexed OCT for multi-spot tracking of induced transient corneal dynamics
Source: Biomed Opt Express. 2026 Apr 29;17(5):2715–33. doi: 10.1364/BOE.596342 (PMC13178628; doi:10.1364/BOE.596342)
Supplement: Supplementary file 1 [file boe-17-5-2715-s001.pdf]

Supplemental Document

Biomedical Optics EXPRESS

Single-shot, depth-encoded multiplexed OCT for multi-spot tracking of induced transient corneal dynamics: supplement

KAROL KARNOWSKI,[1,2,*] JADWIGA MILKIEWICZ,[1,2] ONUR CETINKAYA,[1,2] ANGELA PACHACZ,[1,2] EWA MĄCZYŃSKA-WALKOWIAK,[3] PATRYK MŁYNIUK,[4,5] ANDREA CURATOLO,[1,2,6] KAMIL LIŻEWSKI,[1,2] AHMED ABASS,[7] DAWID BORYCKI,[1,2] BARTŁOMIEJ KAŁUŻNY,[4,5] SUSANA MARCOS,[8,9] AHMED ELSHEIKH,[7] IRENEUSZ GRULKOWSKI,[3,5] AND MACIEJ WOJTKOWSKI[1,2]

[1] *International Centre for Translational Eye Research, ul. Skierniewicka 10a, 01-230 Warsaw, Poland*
[2] *Institute of Physical Chemistry, Polish Academy of Sciences, ul. M. Kasprzaka 44/52, 01-224 Warsaw, Poland*
[3] *Institute of Physics, Faculty of Physics, Astronomy and Informatics, Nicolaus Copernicus University in Toruń, ul. Grudziadzka 5, 87-100 Toruń, Poland*
[4] *Department of Ophthalmology, Collegium Medicum, Nicolaus Copernicus University, ul. M. Curie Skłodowskiej 9, 85-094 Bydgoszcz, Poland*
[5] *Institute of Advanced Studies, Nicolaus Copernicus University, ul. Wileńska 4, 87-100 Toruń, Poland*
[6] *Department of Physics, Politecnico di Milano, Piazza Leonardo da Vinci 32, 20133 Milan, Italy*
[7] *Department of Materials, Design and Manufacturing Engineering, School of Engineering, University of Liverpool, Liverpool L69 3GH, UK*
[8] *Instituto de Óptica "Daza de Valdés", Consejo Superior de Investigaciones Científicas, Madrid, Spain*
[9] *Center for Visual Science, The Institute of Optics, Flaum Eye Institute, University of Rochester, New York, USA*
[*] *kkarnowski@ichf.edu.pl*

This supplement published with Optica Publishing Group on 29 April 2026 by The Authors under the terms of the Creative Commons Attribution 4.0 License in the format provided by the authors and unedited. Further distribution of this work must maintain attribution to the author(s) and the published article's title, journal citation, and DOI.

Supplement DOI: https://doi.org/10.6084/m9.figshare.32038821

Parent Article DOI: https://doi.org/10.1364/BOE.596342

Single-shot, depth-encoded multiplexed OCT for multi-spot tracking of induced transient corneal dynamics: supplement

KAROL KARNOWSKI,[1,2,*] JADWIGA MILKIEWICZ,[1,2] ONUR CETINKAYA,[1,2] ANGELA PACHACZ,[1,2] EWA MĄCZYŃSKA-WALKOWIAK,[3] PATRYK MLYNIUK,[4,5] ANDREA CURATOLO,[1,2,6] KAMIL LIŻEWSKI,[1,2] AHMED ABASS,[7] DAWID BORYCKI,[1,2] BARTŁOMIEJ KAŁUŻNY,[4,5] SUSANA MARCOS,[8,9] AHMED ELSHEIKH,[7] IRENEUSZ GRULKOWSKI,[3,5] AND MACIEJ WOJTKOWSKI[1,2]

[1]*International Centre for Translational Eye Research, ul. Skierniewicka 10a, 01-230 Warsaw, Poland*
[2]*Institute of Physical Chemistry, Polish Academy of Sciences, ul. M. Kasprzaka 44/52, 01-224 Warsaw, Poland*
[3]*Institute of Physics, Faculty of Physics, Astronomy and Informatics, Nicolaus Copernicus University in Toruń, ul. Grudziadzka 5, 87-100 Toruń, Poland*
[4]*Department of Ophthalmology, Collegium Medicum, Nicolaus Copernicus University, ul. M. Curie Skłodowskiej 9, 85-094 Bydgoszcz, Poland*
[5]*Institute of Advanced Studies, Nicolaus Copernicus University, ul. Wileńska 4, 87-100 Toruń, Poland*
[6]*Department of Physics, Politecnico di Milano, Piazza Leonardo da Vinci 32, 20133 Milan, Italy*
[7]*Department of Materials, Design and Manufacturing Engineering, School of Engineering, University of Liverpool, Liverpool, L69 3GH, UK*
[8]*Instituto de Óptica "Daza de Valdés", Consejo Superior de Investigaciones Científicas, Madrid, Spain*
[9]*Center for Visual Science, The Institute of Optics, Flaum Eye Institute, University of Rochester, New York, USA*
**kkarnowski@ichf.edu.pl*

1. Spatial distribution of the sampling points

A recent study of corneal geometry in a population of 2,052 left eyes and 2,135 right eyes diagnosed with KC revealed that the center of the KC cone was most frequently located in the inferior-temporal quadrant [1] [9]. This observation was used to develop 600 KC and 50 healthy eye models using finite-element-method (FEM) under the assumption of a normal intraocular pressure (IOP) of 15 mm Hg [2-3]. During the modeling, we varied the central corneal thickness (CCT) from 450 to 650 µm in 50 µm increments, KC cone center localization (0.0, 0.5, 1.0, 1.5 mm from the apex), KC cone radius (1.5, 2.0 mm), and age (30 to 70 in 10 years increments), and calculated corneal response to air-puff stimulus. The spatial layout that was most effective for asymmetry assessment using eight peripheral spots positioned circumferentially 1.1 mm from the center, and a ninth at the corneal apex.

2. Air-puff chamber

We retained the safety-validated solenoid motor and piston from a commercial non-contact tonometer (Reichert XPert NCT; Reichert Inc., Buffalo, NY, Fig. S1A-B) but designed a new air-pulse module with a fully re-engineered chamber head to eliminate the optical and mechanical limitations of our earlier OCT air-puff prototypes.

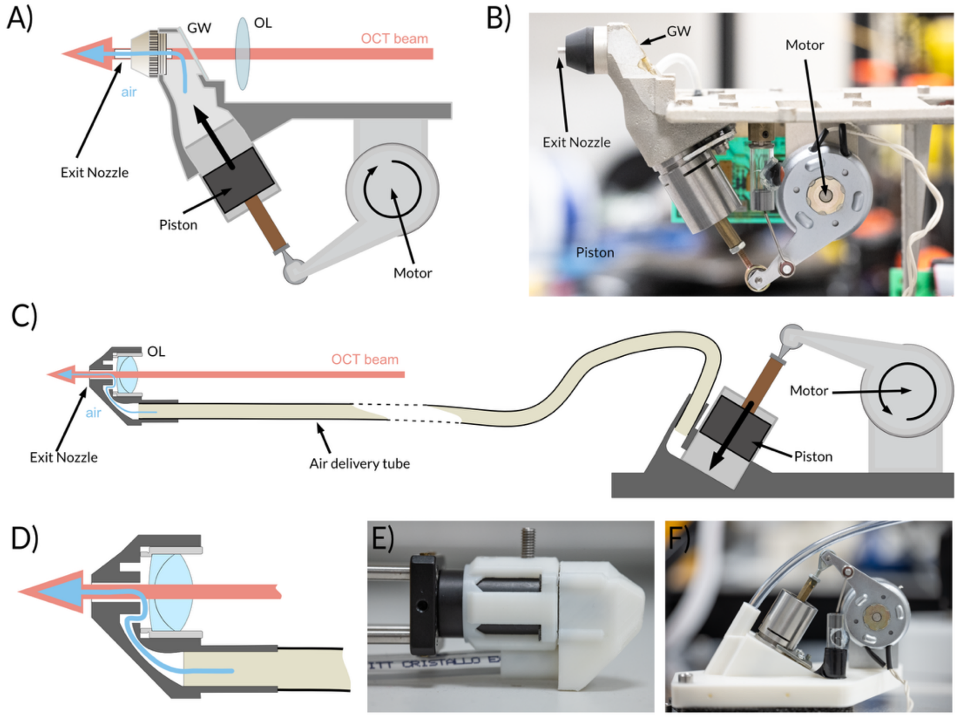

Fig. S1. Air-puff module customization A) Previously used [4-6] air-puff generation module extracted without modifications from commercially available non-contact tonometer (XPert NCT; Reichert Inc., Buffalo, NY). GW – glass window at the rear side of the air-puff chamber, OL – objective lens. B) A photograph of the commercial air-puff generation module. C) Schematic of the modified air-puff generation module where the dynamic part (motor and piston) is separated from the air-puff chamber head. D) Zoomed view on the air-puff head schematic. E) A photograph of the constructed air-puff chamber head with an air delivery tube attached. F) A photograph of the motor and piston part.

The main roles of the air-puff chamber custom design were: (i) to ensure sufficient imaging quality for the peripheral beams, as the original chamber design resulted in significant power losses in both light delivery and collection paths; (ii) to simplify alignment within the nine-spot imaging optics, and (iii) to enable future system miniaturization. The head, machined to fit a

0.5-inch optics footprint and connected to the remote piston through a 5-mm-diameter silicone tube (Fig. S1C), includes a 1300 nm anti-reflection-coated objective lens that replaces the original tonometer's uncoated glass window (Fig. S1D). The AR coated lens improves delivery and collection efficiency – especially for peripheral OCT beams. The separate chamber head (Fig. S1E) isolates the head from piston (Fig. S1F) to minimize piston-induced vibrations. To meet objective (i) and preserve peripheral-beam power, we also widened the exit nozzle diameter. Initial designs and bench tests proved that a bore wider than the factory aperture is necessary to transmits the peripheral beams with negligible loss.

Because the changed bore size might impact the air pulse profiles, we mapped pulse pressure in time (200 µs resolution) and space (0.5 mm step) in front of the nozzle with a WPS500X (Pico Technology Ltd.) pressure sensor mounted on motorized translation stage.

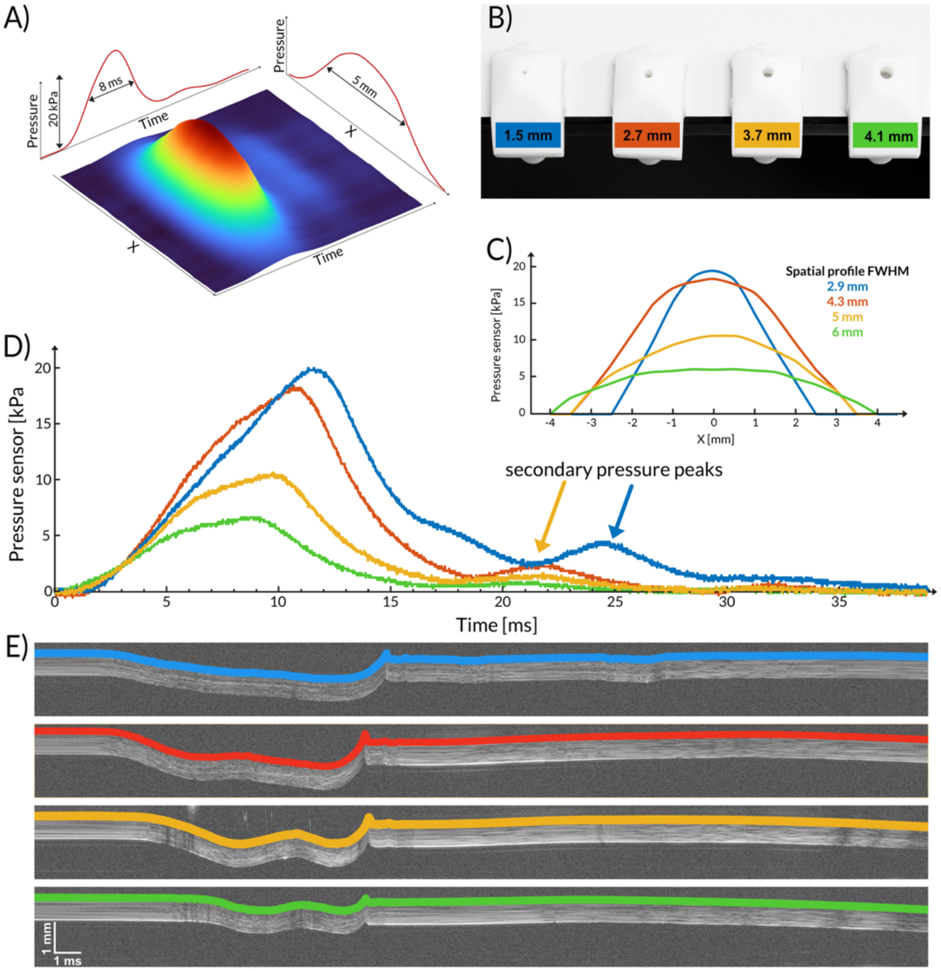

Fig. S2. Investigation of dual corneal indentation effect. A) Example of spatio-temporal (over X axis and time) characterization of the air pulses used for corneal excitation. B) A photograph of the front part of the new air-puff head with 4 different exit aperture sizes used in experiments. C) Spatial profiles (measured along X axis) of the air pulses measured for various sizes of the exit aperture. D) Air-puff temporal profiles measured for various sizes of the exit aperture. E) Air-puff-induced corneal displacement measured with OCT at the cornea apex of the same human subject for various sizes of the exit aperture with overlaid anterior surface of the cornea during air-induced deformation. Colors on plots correspond to measurements with various sizes of the exit aperture (see B and E).

Evolution of the spatial profile of air-puff pressure is presented in Fig. S2A. Quasi-Gaussian distribution of the pressure was observed. As the exit nozzle diameter increased from 1.5 to 4.1 mm, the spatial FWHM broadened from 2.9 to 6 mm (Fig. S2C). Simultaneously, the peak pressure decreased from 20 kPa to 6.5 kPa, whereas the temporal full-width at half-maximum remained ~8 ms (Fig. S2C–D), yielding pulses that are slightly shorter yet substantially spatially wider than those of the unmodified tonometer (compare Fig. S2C and D).

The customized air-puff stimulus led to a novel dual-corneal-indentation response—two distinct displacement events within a single pulse—that, to our knowledge, has not been reported previously (Fig. S2E – third row image with orange profile overlay)). We further explored the effect of bore size on corneal behavior, extending earlier work in which pulse shape had been tuned only by solenoid voltage [7]. Utilizing a nozzle with a 1.5-mm exit aperture, we observed the anticipated single-indentation dynamics in corneal response (depicted in Fig. S2E, blue color). Conversely, for nozzles with larger exit aperture, mentioned dual-indentation effect became apparent. With a 2.7-mm diameter nozzle, the dual-indentation profile exhibited slight peak amplitude asymmetry (Fig. S2E, red color). Moreover, as the nozzle size increased, the dual-indentation dynamics displayed a symmetric displacement amplitude, with a temporal asymmetry in the appearance of these amplitudes (Fig. S2E, orange and green color). Since the width of the pulse spatial distribution is the only air-pulse parameter that is changing with the exit aperture of the nozzle, we hypothesize that this spatial widening of pulse distribution causes the dual-indentation phenomena. Further dedicated research is needed to elucidate the underlying biomechanical mechanisms of this phenomenon.

3. Patient alignment

Precise beam positioning was critical to avoid systematic errors in asymmetry measurements. The central channel is scanned during alignment by the two-axis galvanometer system and therefore appears as a B-scan-like structure (bottom part of Fig. S3B-C and Fig, S3D-E, whereas the peripheral channels are not scanned during alignment and appear as stationary point signals. During the final measurement, all channels are stationary. The operator translated the imaging head (Fig. S3A) in three dimensions until specular reflections—indicating perpendicularity to the central beam in respect to corneal apex—were visible in the center of sequential horizontal and vertical OCT B-scans (Fig. S3B, zoomed in Fig. S3D). Preview scans were saved for verification; if patient movement resulted in a misaligned stimulus (indicated by missing or asymmetric reflection signals, Fig. S3C and S3E), the measurement was excluded and repeated.

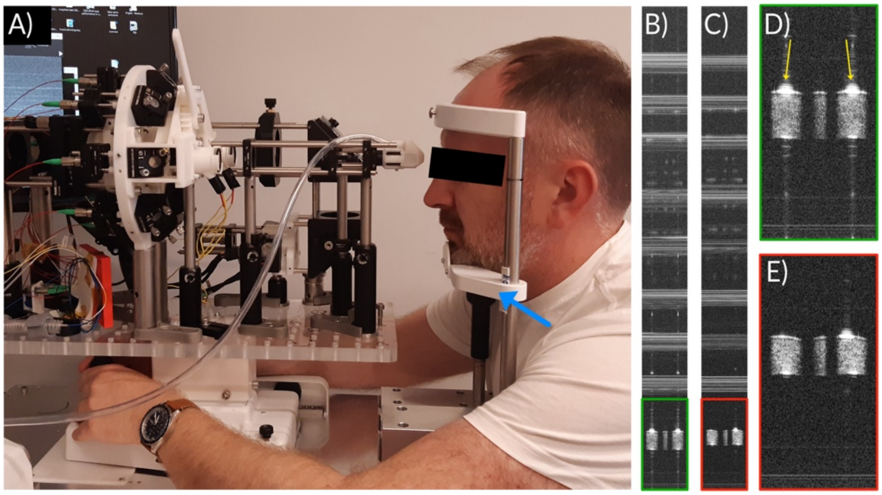

Fig. S3. Subject alignment. (A) Photograph of a subject positioned on the chin rest platform (blue arrow). (B) and (C) OCT preview images demonstrating properly aligned and misaligned cases, respectively. Horizontal patterns represent stationary point images for peripheral spots (not scanned during alignment). (D–E) Magnified regions from the preview OCT images showing the central channel (indicated by green and red boxes in C). Yellow arrows in (D) indicate the specular reflection from the corneal apex.

4. Preselection of optimal parameter of corneal displacement

Because no prior assumption was made regarding which extracted parameter would be the most informative, we compared asymmetry-vector behavior across the full set of derived metrics. DA_1 appeared promising already at the initial stage of this comparison because the difference in asymmetry-vector magnitude between keratoconic and healthy eyes was larger than for the other tested parameters, while the vector direction also showed clinically meaningful agreement (Fig. S4 – first column). This suggested that the DA_1-based vector magnitude could be a useful candidate metric for separating healthy and keratoconic eyes, and DA_1 therefore served as a practical reference for benchmarking the remaining parameters. Some other parameters also showed potentially interesting behavior: for example, DA_3 sometimes yielded vector directions similar to DA_1 (Fig. S4 – second column), but with generally smaller keratoconus-versus-healthy differences in vector magnitude; similarly, the mean value of the DA_1–DA_3 pair showed the same limitation (Fig. S4 – third column). In addition, for the first keratoconic eye, t_{DA2} showed very good directional agreement with the MS-39 maps (Fig. S4 – first row and second last column), although this was not reproduced in the remaining two keratoconic cases. Overall, DA_1 emerged as the most promising single-parameter candidate in the present dataset.

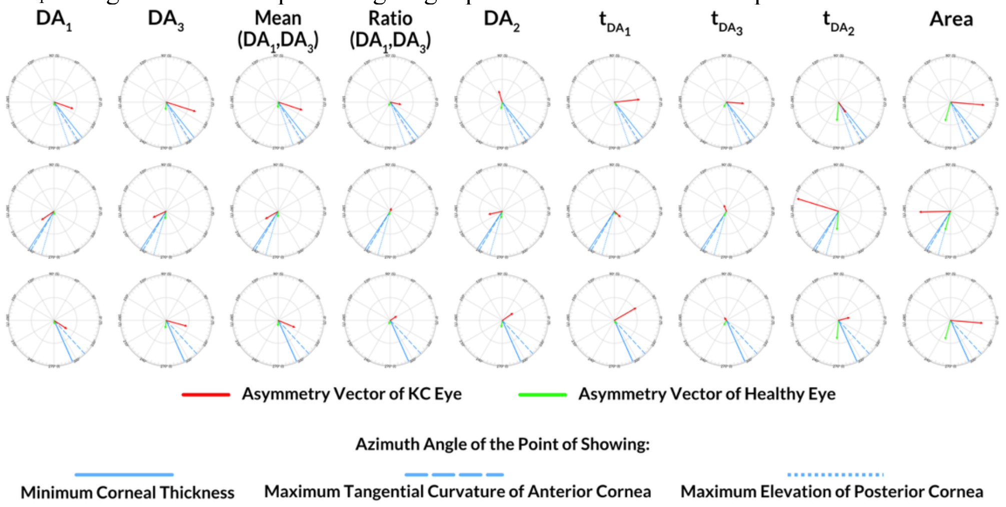

Fig. S4. Asymmetry vectors calculated for additional deformation parameters in the three keratoconus eyes, shown in the same order as in Fig. 6 of the main manuscript. For each parameter, the green arrow indicates the reference asymmetry vector obtained for a healthy eye. Colored azimuth markers indicate the angular position of the abnormal sector derived from the MS-39 maps: minimum corneal thickness (light blue solid line), maximum tangential curvature (light blue dotted line), and maximum posterior elevation (light blue dashed line).

5. Keratoconus phantom

To develop a KC phantom on ex vivo porcine cornea, we used Liberase HI (20 mg/ml) enzyme mixed in a 1:1 ratio with a 30% dextran solution, which was applied for three hours [7-8]. The application of Liberase was aimed at enzymatically degrading collagen fibers within the corneal tissue, anticipated to reduce its biomechanical stability and stiffness. A metal washer restricted the collagenase working area (Fig. 5A), thus enabling local application of the enzyme. The washer was removed before the air-puff OCT measurements. Multi-spot OCT equipped with

an air-puff stimulus was employed to gauge corneal response pre- and post-application of the enzyme. Consistency between sequential measurements was achieved by immobilizing the specimen in a tailored 3D-printed ocular mount. Intraocular pressure was maintained at 15 mmHg via a water column, connected through a needle inserted into the posterior chamber, facilitated by an aperture in the rear of the ocular mount.

6. Temporal down-sampling

The down-sampling process involved extracting subsets of A-scans spaced by the target time interval. This approach yields multiple possible temporal phases for each simulated resolution (e.g., for a 20 µs period simulated from 10 µs data, there are two possible interleaved time series; for a 33.3 µs period there are three possible time series – Fig. S5A-B; for 250 µs, there are 25 possible interleaved time series).

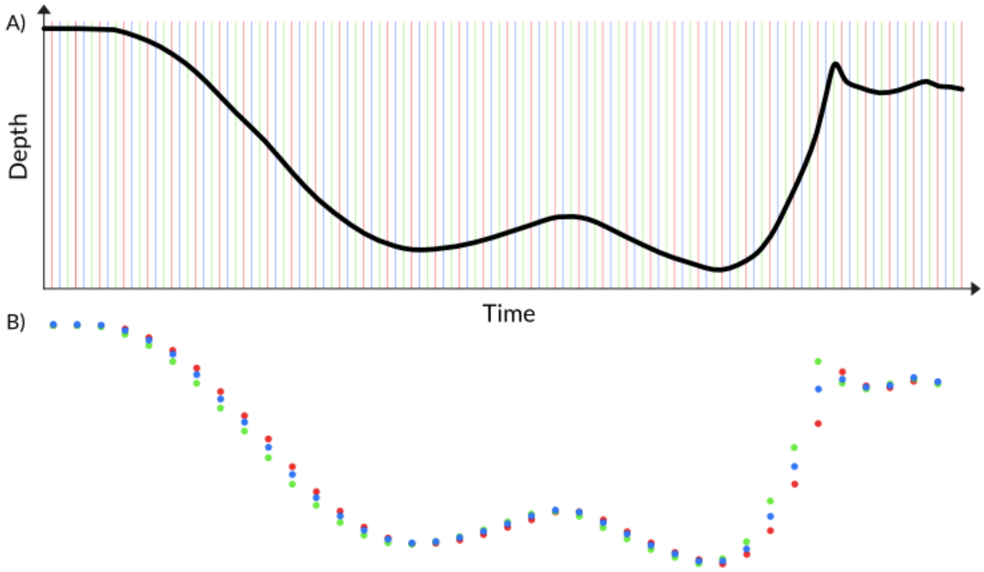

Fig. S5. Graphical explanation of post-processing temporal down-sampling. A) Representative corneal displacement profile at 33.3 µs temporal resolution. The red, blue, and green vertical lines indicate three distinct temporal sampling phases. B) Overlay of the representative corneal displacement profiles sampled at 33.3 µs. This reveals variations in temporal dynamics, demonstrating how sampling differences can lead to errors in extracted parameters (e.g., DA_1) and the resulting asymmetry vector analysis.

References

1. A. Eliasy, A. Abass, B. T. Lopes, R. Vinciguerra, H. Zhang, P. Vinciguerra, R. Ambrósio, C. J. Roberts, and A. Elsheikh, "Characterization of cone size and centre in keratoconic corneas," J. R. Soc. Interface **17**(169), (2020).
2. E. Ahmed, "Finite element modeling of corneal biomechanical behavior," J. Refract. Surg. **26**(4), 289–300 (2010).
3. D. Bronte-Ciriza, D. Bronte-Ciriza, D. Bronte-Ciriza, D. Bronte-Ciriza, J. S. Birkenfeld, J. S. Birkenfeld, J. S. Birkenfeld, A. de la Hoz, A. Curatolo, A. Curatolo, A. Curatolo, J. A. Germann, L. Villegas, A. Varea, E. Martínez-Enríquez, S. Marcos, and S. Marcos, "Estimation of scleral mechanical properties from air-puff optical coherence tomography," Biomedical Optics Express, Vol. 12, Issue 10, pp. 6341-6359 **12**(10), 6341–6359 (2021).
4. D. Alonso-Caneiro, K. Karnowski, B. Kaluzny, A. Kowalczyk, and M. Wojtkowski, "Assessment of corneal dynamics with high-speed swept source Optical Coherence Tomography combined with an air puff system," Opt. Express **19**(15), 14188 (2011).
5. E. Mączyńska, K. Karnowski, K. Szulzycki, M. Malinowska, H. Dolezyczek, A. Cichanski, M. Wojtkowski, B. Kaluzny, and I. Grulkowski, "Assessment of the influence of viscoelasticity of cornea in animal ex vivo model using air-puff optical coherence tomography and corneal hysteresis," J. Biophotonics **12**(2), e201800154 (2019).

6. K. Karnowski, E. Mączyńska, M. Nowakowski, B. Kałużny, I. Grulkowski, and M. Wojtkowski, "Impact of diurnal IOP variations on the dynamic corneal hysteresis measured with air-puff swept-source OCT," Photonics Lett. Pol. **10**(3), 64–66 (2018).
7. A. Curatolo, J. S. Birkenfeld, E. Martinez-Enriquez, J. A. Germann, G. Muralidharan, J. Palací, D. Pascual, A. Eliasy, A. Abass, J. Solarski, K. Karnowski, M. Wojtkowski, A. Elsheikh, and S. Marcos, "Multi-meridian corneal imaging of air-puff induced deformation for improved detection of biomechanical abnormalities," Biomedical Optics Express, Vol. 11, Issue 11, pp. 6337-6355 **11**(11), 6337–6355 (2020).
8. C. W. Hong, A. Sinha-Roy, L. Schoenfield, J. T. Mcmahon, and W. J. Dupps, "Collagenase-Mediated Tissue Modeling of Corneal Ectasia and Collagen Cross-Linking Treatments," Invest. Ophthalmol. Vis. Sci. **53**(4), 2321–2327 (2012).
